# Detecting opinion spams through supervised boosting approach

**DOI:** 10.1371/journal.pone.0198884

**Published:** 2018-06-11

**Authors:** Mohamad Hazim, Nor Badrul Anuar, Mohd Faizal Ab Razak, Nor Aniza Abdullah

**Affiliations:** 1 Department of Computer System and Technology, Faculty of Computer Science and Information Technology, University of Malaya, Kuala Lumpur, Malaysia; 2 Faculty of Computer Systems & Software Engineering, University Malaysia Pahang, Lebuhraya Tun Razak, Gambang, Kuantan, Pahang, Malaysia; Tampere University of Technology, FINLAND

## Abstract

Product reviews are the individual’s opinions, judgement or belief about a certain product or service provided by certain companies. Such reviews serve as guides for these companies to plan and monitor their business ventures in terms of increasing productivity or enhancing their product/service qualities. Product reviews can also increase business profits by convincing future customers about the products which they have interest in. In the mobile application marketplace such as Google Playstore, reviews and star ratings are used as indicators of the application quality. However, among all these reviews, hereby also known as opinions, spams also exist, to disrupt the online business balance. Previous studies used the time series and neural network approach (which require a lot of computational power) to detect these opinion spams. However, the detection performance can be restricted in terms of accuracy because the approach focusses on basic, discrete and document level features only thereby, projecting little statistical relationships. Aiming to improve the detection of opinion spams in mobile application marketplace, this study proposes using statistical based features that are modelled through the supervised boosting approach such as the Extreme Gradient Boost (XGBoost) and the Generalized Boosted Regression Model (GBM) to evaluate two multilingual datasets (i.e. English and Malay language). From the evaluation done, it was found that the XGBoost is most suitable for detecting opinion spams in the English dataset while the GBM Gaussian is most suitable for the Malay dataset. The comparative analysis also indicates that the implementation of the proposed statistical based features had achieved a detection accuracy rate of 87.43 per cent on the English dataset and 86.13 per cent on the Malay dataset.

## Introduction

With technology advancement, the number of people using mobile applications are increasing throughout the world, regardless of the platforms. This increase in mobile use also increases the development of mobile application marketplaces [1]. As is understood, every mobile platform operating system has its own mobile application marketplace for instance, Google Playstore for Android and Apple Appstore for iOS. All these mobile application marketplaces host millions of “free” and “paid” mobile applications, comprising all sorts of categories and languages. The number of applications inside the mobile application marketplaces have exponentially increased between 2009 until 2017 and currently, it carries a record of 2.8 million available applications [2]. These applications get downloads every day by mobile phone users for various usage. There are free applications and there are paid applications. Before purchasing their desired applications, users sometimes like to review the feedback of previous customers to help them make decisions before clicking on the “Purchase” button. Thus, it can be deduced that the feedback and opinions drawn from previous users can contribute significantly to the customers’ decision to make or not make the purchase [3], particularly when high cost applications are involved.

Unlike fact-based decision making which uses facts to arrive at a decision, opinion-based decision making uses others’ opinions or judgements to make selections between the alternatives provided. Often, the opinion of others is considered more highly than the consideration given to the fact of the alternatives [4]. Although there are hundreds of legitimate opinions (i.e. negative or positive) available, some of them are fake, called spam opinions and they exist to confuse the users’ decision. In the case of mobile applications, developers and marketplace providers are constantly challenged by the presence of opinion spams which disrupt their business [5]. Moreover, the presence of fake opinions can be harmful to businesses and result in profit losses [6, 7].

This phenomenon raises concerns in the opinion mining domain whose job it is to detect and filter any review spams [8]. Some of the early approaches used in detecting opinion spams were based on manual judgements but this has become outdated because manual judgements are unreliable [9]. The situation has become more dangerous and unpredictable as more and more opinion spams seem to be emerging, making comments about the respective mobile applications. Consequently, it becomes really difficult to differentiate between legitimate and fake opinions and this has triggered a new trend of opinion mining which comes with sentiment analysis using Natural Language Processing (NLP) [10–12] which is able to detect opinion spams automatically.

Prior to that, researchers such as Jindal and Liu [13] implemented a machine learning approach by adopting the logistic regression to detect opinion spams, so did Li, Ott [14], Lin, Zhu [15] and Ren and Ji [16] who likewise, used supervised machine learning to detect opinion spams. However, earlier studies by Ren, Ji [17] and Li, Chen [18] implemented a hybrid approach that combined supervised and semi-supervised machine learning to detect opinion spams. Despite this being so, there were several limitations to the machine learning approach, for example, using too many features, providing less accurate outcomes, poor flexibility and high consumption of computational time.

Aiming to overcome such limitations, this study proposes using a model that contains statistical based features to detect opinion spams noted in language datasets. The model was developed and then evaluated through the supervised boosting approach which includes the XGBoost, GBM Adaboost, GBM Gaussian, GBM Poisson and GBM Bernoulli. The aim was to see if they are efficient in detecting opinion spams noted in multilingual datasets (i.e., English and Malay). For the purpose of this study, statistical based features taken from the review contents as well as some newly recommended statistical based features were then implemented into the dataset to facilitate analysis. It was deduced that this combination in approach is able to produce multiple predictive models that enhance the accuracy in detection. Accordingly, the contributions of this study are as follows:

- Yelp’s English review datasets extracted from Mukherjee, Venkataraman [19] was used as the public dataset in combination with the private Malay review dataset extracted from Google Playstore.
- Focusing only on statistical-based features, this study adopted some features from previous works and added some new features to be used in the model.
- This study focusses on the boosting approach by using different boosting approaches (i.e., XGBoost, GBM Adaboost, GBM Gaussian, GBM Poisson, GBM Bernoulli) to detect the opinion spams.
- Empirical evidence was used to measure the machine learning performance of the detection model.
- A comparative evaluation of the current and newly proposed features was made through the XGBoost to detect the opinion spams of both the public (i.e., English) and private (i.e, Malay) datasets.

The remaining part of this paper is structured as follows: Section 2 presents the related and previous works on opinion spam detection and the boosting approaches. Section 3 discusses data collection, data processing, features generation and selection. Section 4 presents the results and the evaluation of the private dataset along with a comparative analysis of the newly proposed features on the public and private datasets. Section 5 concludes the work. Section 6 explains the limitations and the recommendation for future works.

## Related works

Reviews or opinions regarding user feedback and user satisfaction about certain product contents and contexts are often used by consumers to assess a particular product/service. They can be helpful in assisting consumers with making decisions about their purchase. Product reviews or consumer opinions are used extensively in businesses as a guide for future customers. In the case of mobile applications, reviews made by other users can also be beneficial. Consumer reviews about mobile application marketplaces such as Google Playstore, especially when coupled by star ratings, can serve as indicators depicting the application’s quality for other users. Likewise, manufacturers or producers offering such applications can use these reviews to upgrade or further enhance their products or services. Although reviews may be an advantage to both the users and producers, some reviews come across as opinion spams. Their existence is unwelcomed because they can offset the balance of online businesses and this can be detrimental to business profits and losses. To address this issue, studies [20, 21] have introduced a few ways of identifying these opinion spams.

The advancement of technology had led to the development of online businesses and so they have also become part of the landscape on mobile applications. In today’s world, mobile users are able to do transactions over their mobile phones and among the facilities provided is the application that allows users or producers to post reviews. However, as is common with all activities that human beings create, some of these reviews are opinion spams which are fictitious opinions given by fake users. Ott, Choi [9] focused on fictitious opinions by looking at crowdsourced fake reviews data. They used n-grams based classifiers to detect these spams. This interest was further extended by Mukherjee, Venkataraman [19] who also studied fictitious reviews by comparing the behavioural features of opinion spams with real-life Yelp reviews dataset. They used the Support Vector Machine (SVM) for their detection. Their study showed that the use of behavioural features enhanced the accuracy of their comparisons when compared to just using the n-gram based approach. Nonetheless, they tested their approach by using generated datasets (i.e. fictitious opinions) which were unable to portray spam detection in real-life reviews.

In an attempt to address this limitation and to portray a real-life review, a ranking-based approach was introduced by Fei, Mukherjee [22]. They explored the reviews burstiness in detecting opinion spammers. They adopted the Loopy Belief Propagation (LBP) and Markov Random Field (MRF) approach to study their data. Clearly, their study aimed to differentiate spammers and legitimate users. Likewise, Akoglu, Chandy [23] also used the ranking-based approach to study spam reviews and they proposed a framework called the FraudEagle which explores the network and graph relationships of the reviews. This was done by using an iterative propagation-based approach for the classifications. It was realised that the FraudEagle successfully detected fraud-bots on an online review website. In a separate study, SPEAGLE was introduced by Rayana and Akoglu [24] which uses information gathered from the review metadata such as texts, timestamps, ratings and network information of the reviews to detect spammers. Thus far, few studies have attempted to detect opinion spams by looking at language specific reviews such as English, Malay, Chinese or other foreign languages.

To date, there is a Chinese language review site called DianPing which is currently available for users to access the reviews of products and services. It is also gaining popularity among customers who use it to check for local business reviews. It appears that opinion spams are also common in this review site but it has been a challenge for reviewers and producers alike, to detect these opinion spams. This is because there is no state-of-the-art approach to enable them to perform this assessment. In an attempt to focus on opinion spams in a non-English platform, Xu, Zhang [25] proposed two novel methods: the K-Nearest Neighbor (KNN) and the general graph-based approach as a methodology. Their study focused on detecting collusive spammers in a Chinese review website. Their results showed that the behaviour of the reviewers also contributed in detecting opinion spams. Focusing on the same area of research, Li, Chen [18] introduced the method called Positive-Unlabeled (PU) to detect fake opinions. Here, reviews were collected from the popular Chinese review website, Dianping [26] and the researchers used the supervised learning approach to detect the fake and unknown reviews. While fake reviews are fictitious, unknown reviews could be fake or genuine. To be on the safe side, the researchers decided to treat the unknown reviews as an unlabelled dataset [27]. Consequently, this approach became known as the PU learning approach. Another study was extended from this by Ren, Ji [17] who proposed using a novel semi-supervised model called the mixing population and the individual property technique which applied to the PU learning (MPIPUL) approach. It was apparent that the PU learning worked well in balanced datasets and thus far, it has not been tested on imbalanced datasets.

Since existing approaches for opinion spam detection suffer from imbalanced datasets, it is most likely that the outcome gathered would be less reliable. Thus Heydari, Tavakoli [28] introduced an effective spam review detection approach that uses deviation of review rating, activeness of the reviewers and content-based information by using time series. This method, however, suffers from expensive computations processing time. In addition, these approaches are less efficient in interpreting the semantic meanings and information that are contained inside the review texts. Ren and Ji [16] thus proposed using the neural network model which combines the convolutional neural network (CNN) and the recurrent neural network (RNN) together in order to learn about the continuous document level representations of the reviews. Their study showed that the neural model has a better generalisation ability as compared to the discrete models.

Spammer detection involves detecting the users’ accounts or profiles that posted the spam contents for malicious purposes. Some detection techniques have used n-gram, some have used linguistics and pattern-based features but a highly-trained spammer able to avoid detection by carefully craft their reviews to make it look genuine. Hence Wang, Xie [29] proposed the idea of using heterogeneous graphs to find and correlate the relationship that is present between the reviewers and the reviews. This approach does not use any text information taken from the reviews and it complements the previous approach thereby, increasing the chance of identifying opinion spammers. The graph-based method was also used by Xu, Zhang [25] in their work. Meanwhile, Ye and Akoglu [30] proposed a two-step method to detect opinion spammer’s groups and products by focussing on network footprints. This technique contains two major modules which are Network Footprint Score (NFS) and GroupStrainer. The experiments found that their approach outperformed existing methods that had studied Amazon and iTunes datasets. This approach also gave a high accuracy rate of the spam detection. Even though there are numerous opinion spams and spammer detection techniques, there are still several limitations in terms of complexity, computational costs and number of features.

Recently, the neural network model has been widely used to detect opinion spams [16, 31]. However, it is known to contain high model complexity due to the high level of details of the neural network. The higher the level of model complexity is, the higher the computational cost of the neural network models [32] and this increases the amount of resources that need to be used for the model adoption. In addition, Heydari, Tavakoli [28] proposed a time series approach to detect opinion spams but it was noted that the method suffered from a high computational problem during the scoring phase. These issues thus restrict the performance of the approaches used for detecting opinion spams as only suspicious intervals are being analysed. In that regard, an additional approach is needed to improve the accuracy of detecting opinion spams.

There are numerous types of classifiers ranging from tree-based, regression, boosting and ensemble to the sophisticated deep neural network architecture. Boosting approaches are currently on the rise among researchers with other popular classifiers being used for solving the classification and regression problems.

### Boosting

Boosting algorithm was initially introduced by Schapire [33] and it portrayed the idea of converting weak learning algorithms into an algorithm with high accuracy. The work was followed up by Friedman, Hastie [34] and Friedman [35] who then made boosting popular by utilising it as an approach for the functional approximation of the logistic regression model. This reliable approach for solving many regression and classification problems is known as the gradient boosting machine. A minor modification was made later by Friedman [36] who adopted the fitting of random subsamples of training sets without replacements. This modification is known as stochastic gradient boosting and it was inspired by the bootstrap aggregating (bagging) method noted in Breiman [37]. The residuals in the approach were based on the minimisation of the loss function gradient and the stochastic gradient descent in regression. Boosting is popular for its speed in building models and its robustness for prediction. Various boosting systems are available for use in various programming and scripting languages. Table 1 shows the comparison of major boosting systems based on their features.

**Table 1:** Comparison of major boosting systems [38].

System	Exact greedy	Approximate global	Approximate Local	Out-of-core	Sparsity aware	Parallel
XGBoost	Yes	Yes	Yes	Yes	Yes	Yes
R GBM	Yes	No	No	No	Partially	No
pGBRT	No	No	Yes	No	No	Yes
Spark MLLib	No	Yes	No	No	Partially	No
Scikit-learn	Yes	No	No	No	No	No
H20	No	Yes	No	No	Partially	Yes

**Fig 1:**
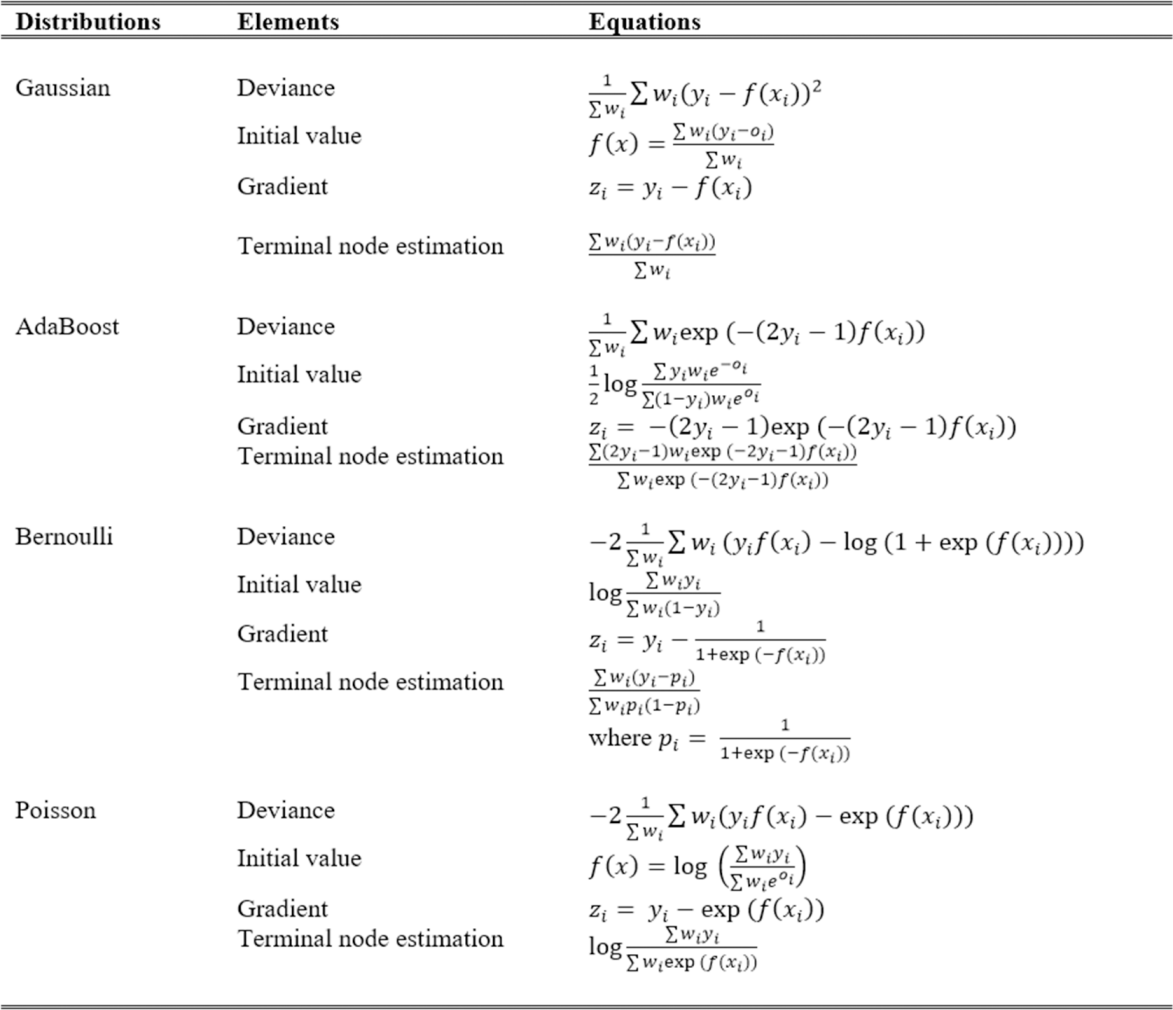
Different flavors of GBM distributions.

It can be seen that the Generalised Boosted Regression Model (GBM) was applied in Table 1 as a boosted regression classifier package in the R statistical software [39]. The GBM package used the approach proposed by Friedman [35] with some modifications made on the implementation. This external package implements the AdaBoost’s exponential loss function approach that was proposed by Freund and Schapire [40] who combined it with the gradient descent algorithm developed by Friedman [35]. The GBM package has various implementations of distributions including Gaussian, AdaBoost, Bernoulli and Poisson. Every distribution has its own class and method in calculating the initial value, the associated deviance, the gradient and the constants for predicting the terminal node. The distributions used in this study are shown in Fig 1.

**Fig 2:**
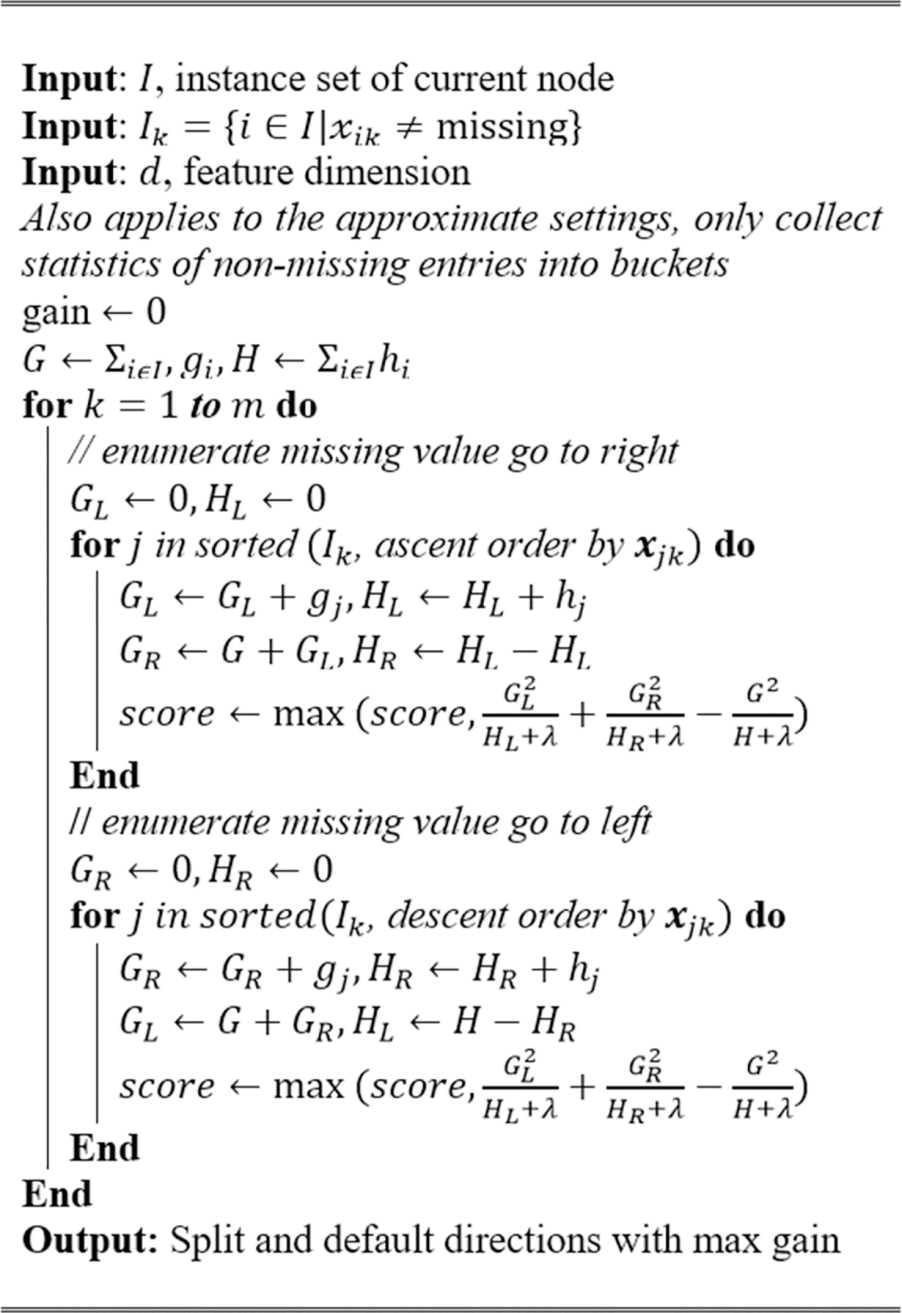
Novel sparsity-aware split finding algorithm [38].

The Extreme Gradient Boosting (XGBoost) is an end-to-end gradient tree boosting system that is scalable and fast in terms of performance [38]. Tree boosting is known for delivering good results in a classification problem [41]. The XGBoost is known for winning various Kaggle competitions and for outperforming other types of classifiers. Only some ensemble classifiers are able to outperform a nicely tuned XGBoost classifier [42]. The essence of the XGBoost lies in the heart of the system. Panda, Herbach [43], Tyree, Weinberger [44] and Ye, Chow [45] initially explored the functionality and effectiveness of a parallel tree boosting system. On top of that, Chen and Guestrin [38] introduced a novel sparsity-aware algorithm for the parallel tree boosting that treats non-presence as a missing value as well as learning the suitable approach to handle the missing values. They then proposed a theoretically justified weighted quantile sketch for an efficient calculation along with a smart cache-aware block structure for out-of-the-core tree learning. Fig 2 shows the novel sparsity-aware split finding algorithm.

Boosting is widely used in various domains to solve classification and regression problems. Persson, Bacher [46] used gradient boosted regression trees to predict the solar power generation on multiple sites. It was found to outperform the existing autoregressive models. Further to that, gradient boosting was also used by Johnson, Ianiuk [47] to predict waste generations across 232 different locations in New York city. The model accurately forecasted the weekly municipal solid waste (MSW) with an average R2 scores of more than 0.88. Pierdzioch, Risse [48] implemented the boosting approach in forecasting the volatility of gold price fluctuations. Similarly, Zięba, Tomczak [49] implemented boosting in the prediction of bankruptcy. The boosting approach has successfully increased the quality of the prediction as compared to other existing techniques. This shows that boosting is increasingly used for solving various classification and regression problems.

This study adopts the boosting approach by using the XGBoost and GBM as the boosting system which is then applied to the domain of opinion spam detection on multilingual datasets. This study also introduces new statistical features to increase the performance of the model in detecting opinion spams in Malay and English language datasets.

## Methodology

**Fig 3:**
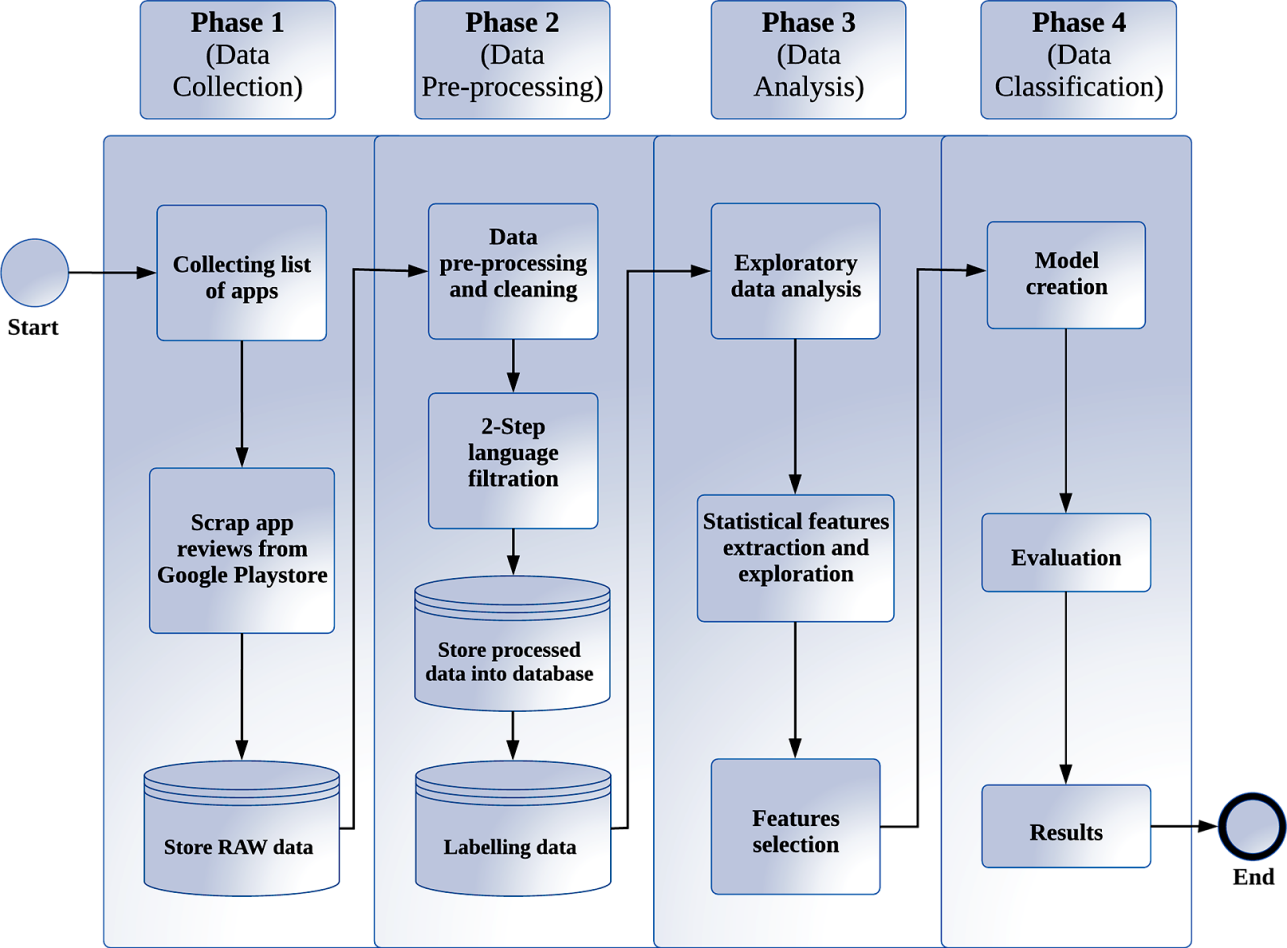
Research methodology workflow.

Aiming to detect opinion spams in mobile application marketplaces of multilingual datasets (i.e., English and Malay), the statistical based features were proposed and then modelled with supervised boosting approaches such as the Extreme Gradient Boost (XGBoost and Generalized Boosted Regression Model (GBM). Fig 3 illustrates the research methodology workflow which is divided into 4 phases: a) data collection, b) data processing, c) data analysis and d) data classification.

### Phase 1 (data collection)

There are two (2) datasets involved: a) public hotel dataset downloaded from Mukherjee, Venkataraman [19] on 16^th^ February 2017 and b) private dataset collected from Google Playstore. The former contains English opinions gathered from the Yelp website (i.e. a commercial review website that hosts reviews for numerous venues such as hotels and restaurants). Since Yelp has its own filtering algorithm to filter spam opinions, it is very interesting to use Yelp’s opinions dataset for the comparative evaluation. The availability of the public dataset for English eases the work on opinion spam detection whereas there is a lack of public dataset for the latter. Opinion spam detection in the Malay language is hard to come by.

Due to the inadequate Malay language public dataset, Google Playstore was accessed on 4^th^ December 2016 by using specific criteria as summarised in Table 2 for the private dataset collection. Random selection of application in Google Playstore was applied and the collection process focussed on applications from Malaysia only. Using a custom scripting, the collection process collected 500 applications and they varied in categories ranging from entertainment, education, games, sports to travels. Using a customised Python HTTP API script, the process of extracting the opinion took 11 hours and a total of 44197 opinions were collected during the process. The collected opinions were then stored in the HTML format. Since there is a lack of published analysis on Malay opinion spam detection, it is worth noting that the current Malay spam opinions dataset are significant for research, especially in the area of opinion spam detection.

**Table 2:** Pre-processed elements from raw data.

Element	Description
url	URL to the app’s playstore page.
appId	App id of the respective app.
title	Name of the app.
summary	Summary description of the app.
developer	Content type of the request
icon	URL to the app’s icon.
score	Average rating of the app
price	App’s price
free	True/False indicator if the app is free or paid.

### Phase 2 (data processing)

Three main processes are involved at this phase: a) data pre-processing and cleaning, b) language filtration and c) labelling. Upon data being collected, the pre-processing was conducted. The cleaning techniques engaged at this phase are important for clearing the unwanted information and other residues from the raw data. In this study, data processing only involved the private dataset because the public dataset was already well processed.

**Fig 4:**
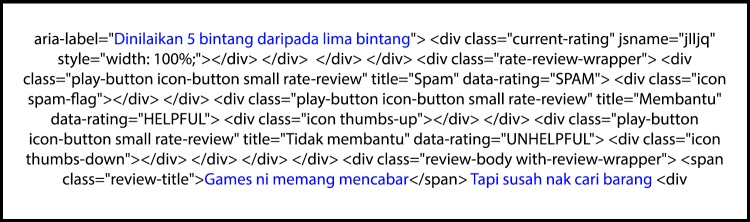
Snippet of raw HTTP response data.

While collecting the reviews and opinions, it was noted that the HTTP POST requested for replies with a raw HTML response. All these raw responses needed to be pre-processed for them to become meaningful data. Every request taken from the opinion extracting session contributed to one HTML raw file which contained a lot of unnecessary information and syntaxes. A snippet of the response is shown in Fig 4.

Fig 4 highlights the important information extracted from the data which are in bold. The pre-processing phase used Python to iteratively extract the desired information from the raw data file based on the important HTML class such as ‘*review-body’* and ‘*aria-label*’ for every review’s HTML div. This pre-processing led to each opinion having its own Javascript Object Notation (JSON) entry. All opinions for a single application were stored in a single JSON file. These files were saved into the local storage for precaution. All the texts were encoded in the UTF-8 for standardisation. Each application has its own JSON file where every entry would store the information, as displayed in Table 3. After saving the data to the local storage, the data then proceed to the cleaning process.

**Table 3:** List of elements extracted from a RAW response file.

Element	Description	Value	Translation (English)
appID	App id of the app.	com.outfit7.talkingpierrefree	com.outfit7.talkingpierrefree
appPrice	Price of the app	0.0	0.0
appScore	Avg rating of the app.	4.2	4.2
appTitle	Name of app.	Talking Pierre the Parrot	Talking Pierre the Parrot
revAuthor	Reviewer’s name.	Maya Liya	Maya Liya
revDate	Date of the review submitted.	14 Mei 2015	14^th^ May 2015
revRating	Rating given by the reviewer.	5.0	5.0
revTitle	Title of review	Best	Best
revText	The review body.	game ni sangat best	This game is so good

As mentioned earlier, since this study has collected textual applications reviews, cleaning the data was essential to make the review readable and easy to process. This study used Python code and regular expressions (regex), a cleaning mechanism that removes all the punctuations that were present on the opinions body and their title. Besides handling punctuations, the process also replaces all missing values in every element of the reviews so as to become NA.

#### Language filtration

**Fig 5:**
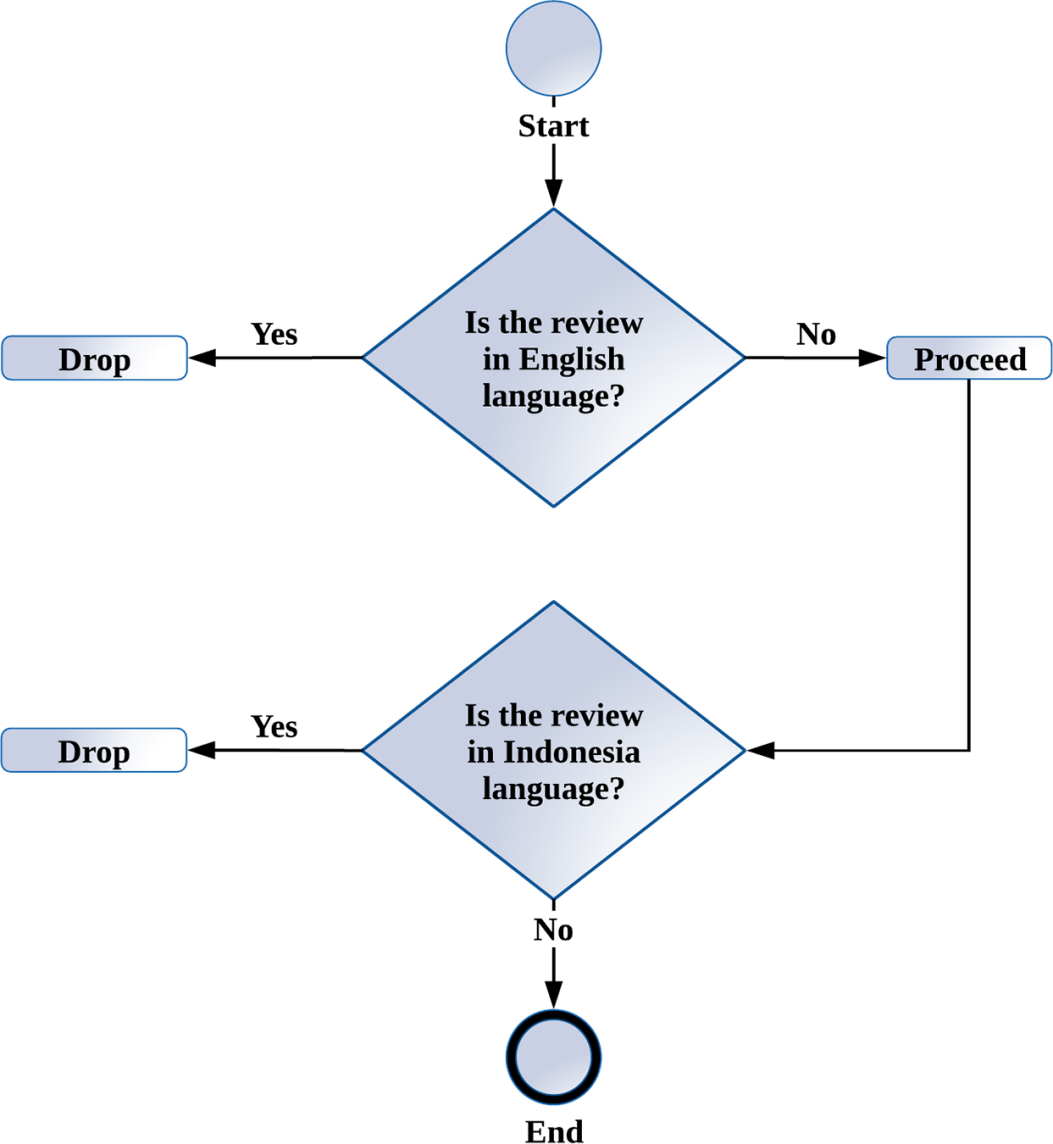
The flow of two-step language filtration process.

Even though this study explicitly defines the parameter for language to be extracted, Google Playstore often mislabelled the application opinions. The reviews extracted from the marketplace were a combination of English, Malay and Indonesian languages. Since the focus of this study was on the Malay opinions, appropriate steps needed to be taken to filter unwanted opinions from the collected data. The process requires a 2-step language filtration mechanism that allows filtering to be done on the English and Indonesian language. Fig 5 depicts the flow of the filtration process.

The filtration process distinguishes the reviews with the correct language through the following steps:

- The review was split into word tokens for comparison.
- A comparison of word tokens in the reviews was performed through pattern-matching detection based on 1028703 English words dictionary.
- A counter calculated the number of English words that matched the respective review.
- If the number of English words matching the counter is equal to a number of words in the review, the system would drop the reviews and move on with the next review.
- Pattern matching is also used to detect any Indonesian words based on 529 mostly used Indonesian words.
- The Indonesian words counter kept track of the number of words matched.
- A threshold was implemented because some Malay opinions mixed the language with some Indonesia language since both languages share some words in common.
- If the number of Indonesian words matched was higher than half of the number of words, the system drops the reviews and proceeds with the next review.

This two-step language filtration mechanism effectively filters out 38639 English reviews and 106 Indonesian reviews from the total of 44197 reviews collected. This filtration process took nearly 10 hours on a normal Core i7 Hewlett Packard laptop with 4GB of RAM. The filter resulted in 5452 reviews left. The filtered data were then saved into a MariaDB database for labeling in the next part.

#### Opinion labelling

The opinion labelling process aims to classify the collected opinions into two categories: a) fake or b) normal. To accomplish this, a simple web framework (i.e. Flask) was used to ease the labeling work. The labelling process was done manually using specific rules as tabulated in Table 4. These rules were based on the works of Sharma and Lin [50] and Geetha, Singha [51] who studied the relationship between customer review sentiments and review ratings. Their findings indicated that there was consistency between review rating and sentiment polarity.

**Table 4:** Rules for labeling reviews.

Review rating	Review sentiment polarity	Label
[0, 1, 2]	Negative	Normal
3	Negative	Spam
[4, 5]	Negative	Spam
[0, 1, 2]	Neutral	Spam
3	Neutral	Normal
[4, 5]	Neutral	Spam
[0, 1, 2]	Positive	Spam
3	Positive	Spam
[4, 5]	Positive	Normal

Apart from using the rules recommended by prior studies, as noted in Table 4, the meaningfulness of the opinions was also considered. This is because some reviews may receive acceptable review ratings and polarity but they were not giving opinions but spamming the review section. In this regard, they were excluded from the opinion dataset. This process resulted in 5000 opinions that consist of 4048 normal and 952 spam opinions to be selected while 452 opinions were dropped. The normal and spam opinions acted as the ground truth for the private dataset.

### Phase 3 (data analysis)

This phase used some data analysis and exploration techniques for the labeled dataset. In the context of this study, analysis of the dataset was done using some notable data analysis techniques. Following this, statistical features were generated and extracted from the dataset to prepare them for feature selection. The feature selection process used the XGBoost features to rank the functions which select the best features that would contribute to the detection model.

#### Exploratory data analysis

An exploratory data analysis was performed on the data collected to uncover any hidden correlations or connections. The exploratory data analysis was done on the labeled dataset using RStudio. The distribution of the reviewers’ sentiment polarity across the datasets showed that of the 5000 opinions selected, 3649 were positive, 550 were neutral and 801 were negative opinions. This study has investigated an interesting distribution by analysing the opinion spams that were in the neutral sentiment polarity category which were from the private dataset, as shown in Table 5. Findings suggest that opinions which were labeled spam and categorised as neutral, mostly consist of questions and unrelated statements. In addition, some reviews also comprised random alphabets, words and sentences.

**Table 5:** Sample of spam reviews in neutral sentiment polarity category in private dataset.

Review author	Review text	Translation (English)	Rating
Aku Ya	cacing ni halal ke haram bro ☺	bro, is this worm halal or haram ☺	5
Amirul Izzat	hmmm	hmmm	5
Nadi Sudin	2016	2016	5
Abu Zaid	pakai internet ke	are you using the internet	5
badri timalsena	Wew	Wew	1
NA	xgdjgokzfbbinovgvkbjjcbjkdvxfp	xgdjgokzfbbinovgvkbjjcbjkdvxfp	5
Ina Evaina	Aku belom coba game ni	I haven’t tried this game	5
Noriha Abf Ghani	Ewr	Ewr	5
Mawar Izuan	hai mawar	hey mawar	1

**Fig 6:**
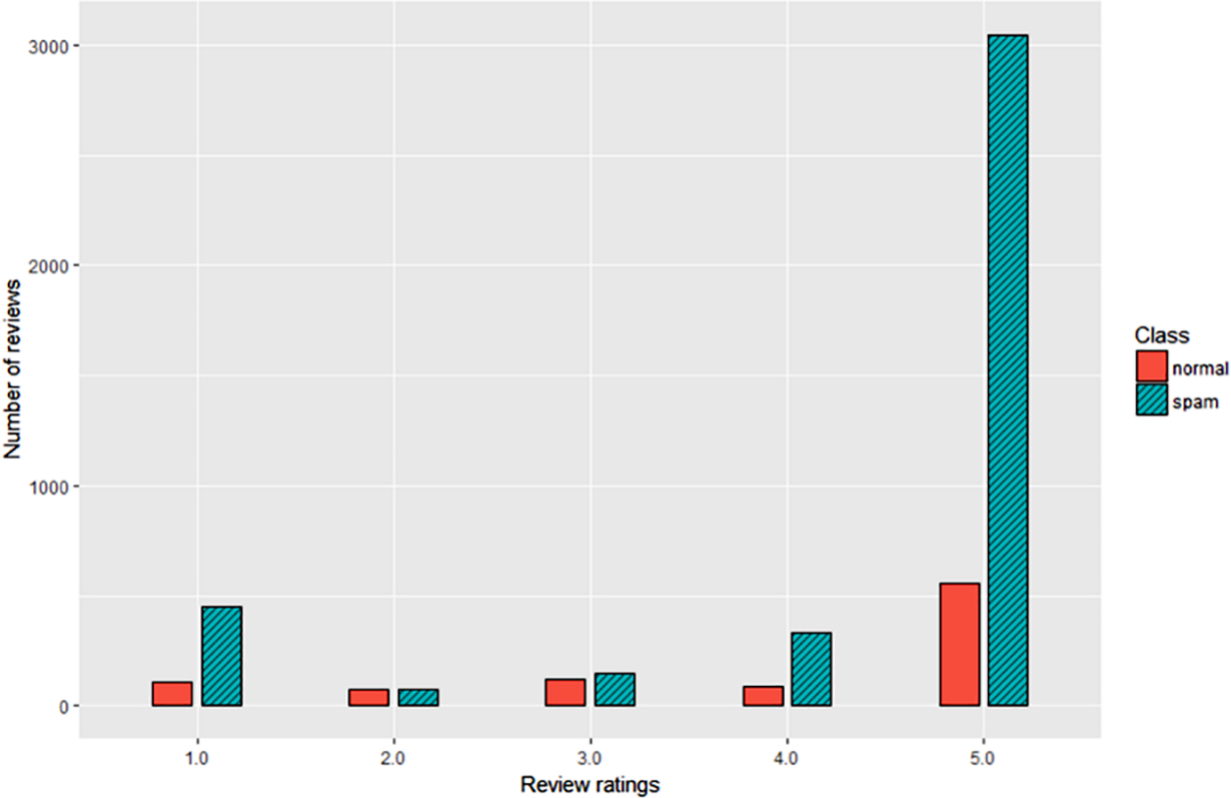
Distribution of spam and normal reviews across review ratings in private dataset.

In the next phase, the distribution of the ratings was examined, across normal and spam opinions, in the private dataset. This was done by creating a grouped bar chart to visualise the distributions as shown in Fig 6. The classes were unevenly distributed across different review ratings as the highest number of spam reviews belonged to review ratings of 5 followed by a rating of 1. This relationship was further investigated and results showed that most of the opinion spammers chose a rating of 1 or 5. It was done to ease their process in the spamming opinions for a certain application, whether it was to popularise or de-popularise the application. Besides that, a review rating of 4 explains that spammers tried to avoid detection by choosing 4 as their preferred ratings because the rating of 5 was too obvious since they faked other elements of the reviews such as author’s name, review titles and review texts.

#### Features extraction and selection

**Fig 7:**
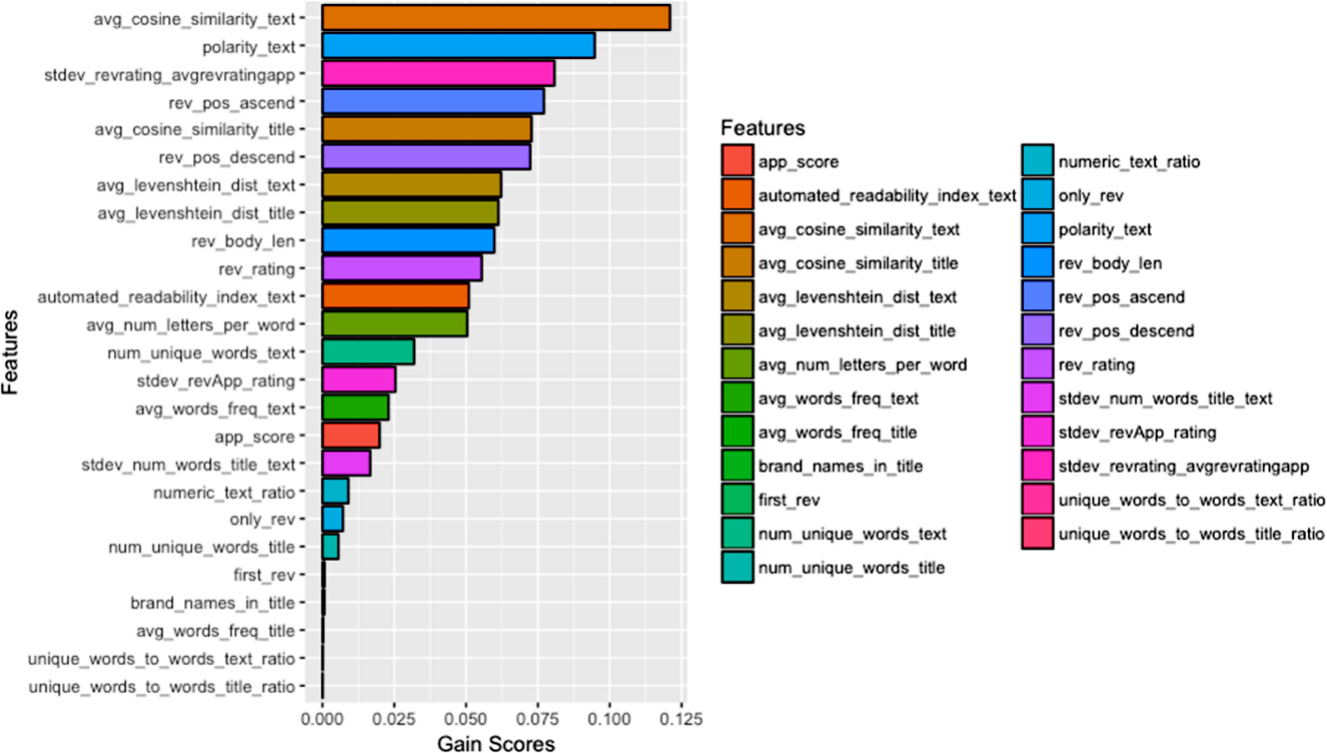
Features ranking based on variable importance gain scores.

At the phase of the features extraction and selection, a total number of 26 features were extracted from the datasets, combination from previously used features along with newly proposed features. However, the number of features actually matters in building a good and reliable predictive model. Besides that, an extra number of features may contribute in overfitting and increase the complexity of the predictive model [52]. This study performed feature selection method by doing a features importance ranking using XGBoost. The variable importance function in XGBoost calculated the gain score of all the features in the dataset along and ranked them based on their importance for making a decision. The gain score of the variable importance was calculated based on how a feature is important in making a branch of decision tree to be purer. Fig 7 shows the ranked features based on their gain score from highest to lowest gain score.

Even though there are 26 features extracted, only some of it are available in the public dataset. This study uses only the features available on both datasets. The top 10 features were selected out of the 26 features to be the final set of features for use in this study. Table 6 shows the list of features used in this experiment.

**Table 6:** List of existing and proposed features.

Label	Features	Category	Gain Score	References
F6	Average cosine similarity between review bodies.	Numerical	0.12102	[15, 20]
F26	Sentiment polarity of review text	Categorical	0.09480	[17, 53]
F4	Position of the review in the reviews of a product sorted by date (ascending).	Numerical	0.07711	[20]
F5	Position of the review in the reviews of a product sorted by date (descending).	Numerical	0.07237	[20]
F2	Length of review body.	Numerical	0.05983	[20]
F3	Rating of review.	Numerical	0.05547	[20]
F15	Automated Readability Index (ARI) of review body.	Numerical	0.05102	[54]
F14	Standard deviation between average review ratings with current review rating.	Numerical	0.08079	Proposed
F7	Average levenshtein distance between review bodies.	Numerical	0.06223	Proposed
F13	Average number of letters per word in review body.	Numerical	0.05044	Proposed

### Phase 4 (data classification)

This phase discussed the model creation process as well as the type of classifiers used to build the prediction model. In the context of this study, separate models were created for each dataset (Malay and English). The final preparation of data was explained together with the tools used for building the predictive model. The model was further evaluated before the results were presented.

#### Boosting algorithm for classification in experiment environment

Predictive models ranged from a simple linear regression approach to a complex neural network approach. Building predictive models require specific tools that support the classifier used. Many tools were available for building the predictive model- from a Graphical User Interface (GUI) software to a library, by the writing scripts. This study used R as the main language in building the predictive model. Since features extraction and generation were done in the RStudio, the resulted private dataset were placed in the form of the data frame. In R, the best way to manipulate, explore and analyse is by converting any table-like document into a data frame. In the context of this study, the datasets were exported into the .csv file for storage and for ease of use in the future by other tools. The English dataset had a total of 2526 opinions taken from Yelp’s hotel reviews [19]. It consisted of 2136 normal and 389 spam opinions. Meanwhile, the Malay dataset had 5000 opinions which consisted of 4048 normal and 952 spam opinions. Both datasets include 11 columns including the class column. Each row in the dataset was a combination of between numerical features and categorical features. The numerical features column was in the format of double-precision floating-point while the categorical features were formatted with one-hot encoding technique which was a technique used to replace a nominal or ordinal categorical value of a column by encoding it with different numbers.

**Fig 8:**
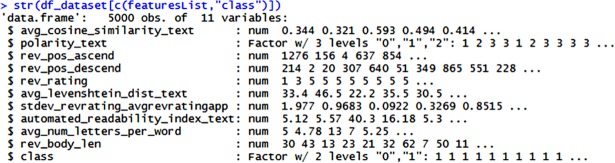
Snippet of the type of features in R data frame for the private and public datasets.

All the features in the dataset were then placed into their respective types. The class column which represented the type of opinions was factorised as level 0 for spam and level 1 for normal. Factorisation separates the categorical features column from the numerical features column in the data frame as shown in Fig 8. Before the datasets were fed into the machine learning classifier, the order of the data was randomised so as to avoid any bias-related elements being considered when building the predictive model. The datasets were randomised multiple times for conformity of randomness.

Machine learning approaches have been widely used in various domains and machine learning approaches tend to focus on making predictions based on a certain target [55]. Boosting is one of the many elements used in machine learning for the creation of a predictive model. In this regard, the XGBoost and GBM package in R were applied for boosting the classifier. The XGBoost was implemented together with different distribution flavours of the GBM and they include Adaboost, Gaussian, Bernoulli, and Poisson. All these boosting machine learning classifiers utilised the data frame prepared in R. Consequently, the data frame was fed into the different machine learning classifiers that utilised the boosting approach to build the predictive models. With that in mind, the 70/30 percent splitting technique of data was applied in terms of the training and testing set. For the training set, the current study randomly allocated 70 per cent of data which were used to train the predictive model. Meanwhile, the remaining 30 per cent of the data were treated as the testing set. They were used to test the detection performance of the predictive model on previously unseen data. The data were randomly sampled and evenly divided based on its class to avoid any imbalanced data distribution. This process was achieved by using a *createDataPartition ()* function that was been adopted from the *caret* package which was available as an external package in R. Accordingly, the training set was used in building all the boosting predictive model. Meanwhile, the test set comprised a hidden testing set; it was applied only once to evaluate the performance of the predictive model.

## Results and evaluations

The proposed features were then evaluated to detect the opinion spams existing in the multilingual datasets. This was accomplished by using supervised boosting approaches. Following this, a comparison was performed by conducting experiments using different sets of features based on the current statistical based features and the newly proposed features. The dataset of the English and Malay reviews were used in this analysis. All the performance metrics used to measure the performance of the predictive model are further discussed.

### Experimental setup

The experiments performed in this study were done on a Mid 2012 Macbook Pro with a 2.5 GHz Intel Core i5 and a 16GB 1600 MHz DDR3 RAM. The operating system used was macOS Sierra 10.12.4 which runs on a 250GB Samsung 850 Evo Solid State Drive (SSD). A choice was made for the different systems of boosting algorithm to be applied as the comparison so as to evaluate the performance of the predictive model in detecting opinion spams in multilingual datasets. The performance of the newly proposed statistical based features was then evaluated using the selected boosting approach. The performance of the predictive model was also evaluated using the multiple performance metrics. Table 7 shows the performance metrics used in the predictive model evaluation for this study.

**Table 7:** Performance metrics used in model evaluation.

Evaluation measure	Descriptions
Confusion matrix	Shows the information about the actual and predicted classifications.
Accuracy	Calculates the percentage of correctly predicted instances either normal or spam.
Sensitivity/ True positive rate (TPR/ Recall	Calculates the correctly predicted instances as spam.
False Positive Rate (FPR)	Calculates the incorrectly predicted instances as spam.
Specificity/ True Negative Rate (TNR)	Measures of correctly predicted instances as normal.
Precision	Measures whether the prediction is precise or not.
F-measure	Calculates the weighted harmonic mean of precision and recall.

### Experimental evaluation and discussions

This study had aimed to detect opinion spams on multilingual datasets by using various supervised boosting approaches. As a comparative study, it evaluated the existing statistical features of different supervised boosting approaches in multilingual datasets. The most suitable approach was selected for the second comparative study. Table 8 shows the comparative evaluation done with different boosting classifiers using existing features on the English and Malay datasets.

**Table 8:** Evaluation of different boosting classifiers using existing features on multilingual datasets.

Dataset	Evaluation measure (%)	XGBoost	GBM AdaBoost	GBM Gaussian	GBM Bernoulli	GBM Poisson
English	Accuracy	85.45	85.19	**85.71**	84.92	85.45
	Recall	**29.31**	12.93	21.55	16.37	5.17
	FPR	4.37	1.72	2.19	2.66	**0.00**
	Specificity	95.62	98.28	97.34	97.34	**100.00**
	Precision	54.84	57.69	59.52	52.78	**100.00**
	F-measure	**38.20**	21.12	31.65	25.00	9.83
Malay	Accuracy	85.20	85.07	**85.27**	84.87	84.87
	Recall	36.53	48.23	**56.38**	36.52	29.43
	FPR	3.53	6.40	8.04	3.94	**2.30**
	Specificity	96.47	93.60	91.95	96.06	**97.70**
	Precision	70.55	63.55	61.87	68.21	**74.77**
	F-measure	48.13	54.84	**59.00**	47.58	42.24

In comparing the results acquired from the different boosting approaches for the English dataset, it was found that the GBM Gaussian achieved the highest accuracy of 85.71 per cent as compared to other approaches. Nonetheless, the recall percentage of the model had determined the true positive rate which showed the rate of accurately detecting the opinion spams. It was also noted that the XGBoost achieved the highest sensitivity with the percentage of 29.31 per cent while the GBM Poisson dominated the evaluation results with the highest value of false positive rate, specificity and precision which are 0 per cent, 100 per cent and 100 per cent respectively. It had successfully classified all the normal opinions without a single false detection. This shows that the model produced by the GBM Poisson has a very high overfitting rate since the recall was 5.17 per cent which is the lowest among all the models. In terms of the f-measure, the XGBoost had achieved the highest percentage of 38.20 per cent as compared to other approaches. The f-measure showed the balance of the results in terms of precision and recall. Since the dataset also contained unbalanced class, the high f-score showed that the model was able to balance the detection of spams and normal opinions. The analysis also showed that the XGBoost was the suitable boosting approach to be used for detecting opinion spams in the English language reviews. However, the results of detecting opinion spams in Malay were different.

The comparison of the different boosting approaches for the Malay dataset showed that the GBM Gaussian had achieved the highest accuracy and recall percentage, which is 85.27 per cent and 56.38 per cent, respectively. The recall percentage implies that the GBM Gaussian works well in detecting the opinion spams by achieving the highest true positive rate. However, in terms of the false positive rate, specificity and precision, the GBM Poisson consistently leads the evaluation result of 2.30 per cent, 97.70 per cent and 74.77 per cent, respectively. These results imply that the model was suffering from overfitting, which is the same case as the English dataset. This outcome shows that the GBM Poisson models were more inclined to learn and fit the noises into the datasets. In terms of the f-measure, the GBM Gaussian also projected an evaluation rate of 59.00 per cent. This indicates the ability of the model to balance the positive rate and the false positive rate. The results and analysis showed that the GBM Gaussian is suitable for detecting opinion spams in the English language dataset.

Based on the detection of the English and Malay opinion spams, it can be deduced that the implementation of a multilingual model had allowed the model to detect both the English and Malay opinion spams. Further, it can also be deduced that the GBM Gaussian has the highest accuracy rate with the second highest being recall and the last being precision scores, as seen in the case of the English dataset. This suggests that the GBM Gaussian is comparable with the XGBoost in creating a multilingual detection model. In looking at the Malay dataset, the GBM Gaussian is also noted to be the most suitable classifier for detecting opinion spams. With some tradeoffs between the detection performance of the GBM Gaussian and the XGBoost in the English dataset combined with the advantage of the opinion spam detection in the Malay dataset, it seems undeniable that the BM Gaussian is the most suitable classifier to be used for training a multilingual detection model. Besides that, the existing set of statistical features was also able to detect the opinion spams in multilingual datasets. It appears that implementing a new set of statistical features increases the performance of the detection model.

This study had adopted several new statistical based features to detect opinion spams in multilingual datasets. The newly proposed features were tested on the multilingual datasets with the most suitable boosting approaches. According to the analysis as shown in Table 8, the XGBoost appears to be suitable for the English language dataset while the GBM Gaussian is suitable for the Malay language dataset. A further evaluation of the implementation of the newly proposed features was also conducted using the aforementioned boosting approaches. Table 9 shows the evaluation results of the existing set of statistical features and the newly proposed features combined together. Table 10 shows the confusion matrix for all the English and Malay models.

**Table 9:** Comparative evaluation with existing and proposed features on English and Malay datasets.

Evaluation measure (%)	Without proposed features (English-A)	With proposed features (English-B)	Without proposed features (Malay-A)	With proposed features (Malay-B)
Accuracy	85.45	**87.43**	85.27	**86.13**
Recall	29.31	**43.97**	56.38	**57.45**
FPR	**4.37**	4.69	8.04	**7.22**
Specificity	**95.62**	95.31	91.95	**92.78**
Precision	54.84	**62.96**	61.87	**64.80**
F-measure	38.20	**51.78**	59.00	**60.90**

**Table 10:** Confusion matrix for all English and Malay models.

Model	Predicted	Actual	
		Fake	Normal
English-A	Fake	34	28
	Normal	82	612
English-B	Fake	51	30
	Normal	65	610
Malay-A	Fake	159	98
	Normal	123	1120
Malay-B	Fake	162	88
	Normal	120	1130

**Fig 9:**
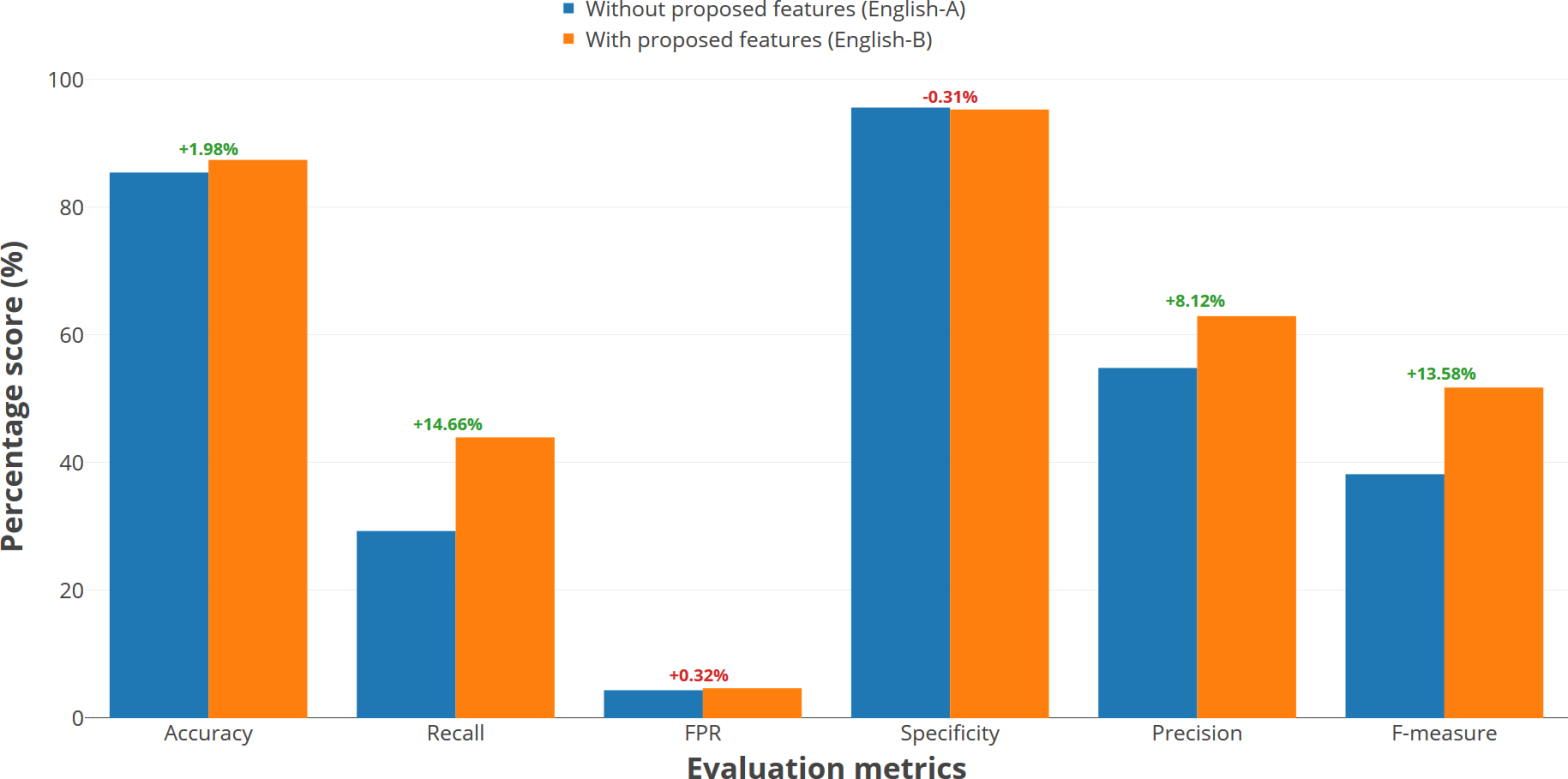
Percentage of score difference between English-A and English-B models.

In evaluating the English dataset through the two models (i.e. English-A and English-B), the proposed features appear to increase the accuracy by 1.98 per cent, moving from 85.45 per cent to 87.43 per cent. The increment of the accuracy also reflects the percentage of the recall scores which was 43.97 per cent, showing a 14.66 per cent increment, when compared to the existing set of features. As seen in Table 10, the number of opinion spams correctly detected had increased from 34 to 51, suggesting that it is the works of the proposed features. This outcome is consistent with the aim of the study. In this regard, the English-B model suffered a marginal difference in terms of false positive rates and specificity when compared to the English-A model. Nonetheless, the English-B model led the comparison in terms of precision and f-measure, with a percentage of 62.96 per cent and 51.78 per cent, respectively. Fig 9 visualizes the percentage of score difference between both English predictive models. It is deduced that the proposed features had increased the f-measure by 13.58 per cent, which is very significant. This shows that it had reduced the overfitting of the model when detecting opinion spams and normal opinions for the English dataset.

**Fig 10:**
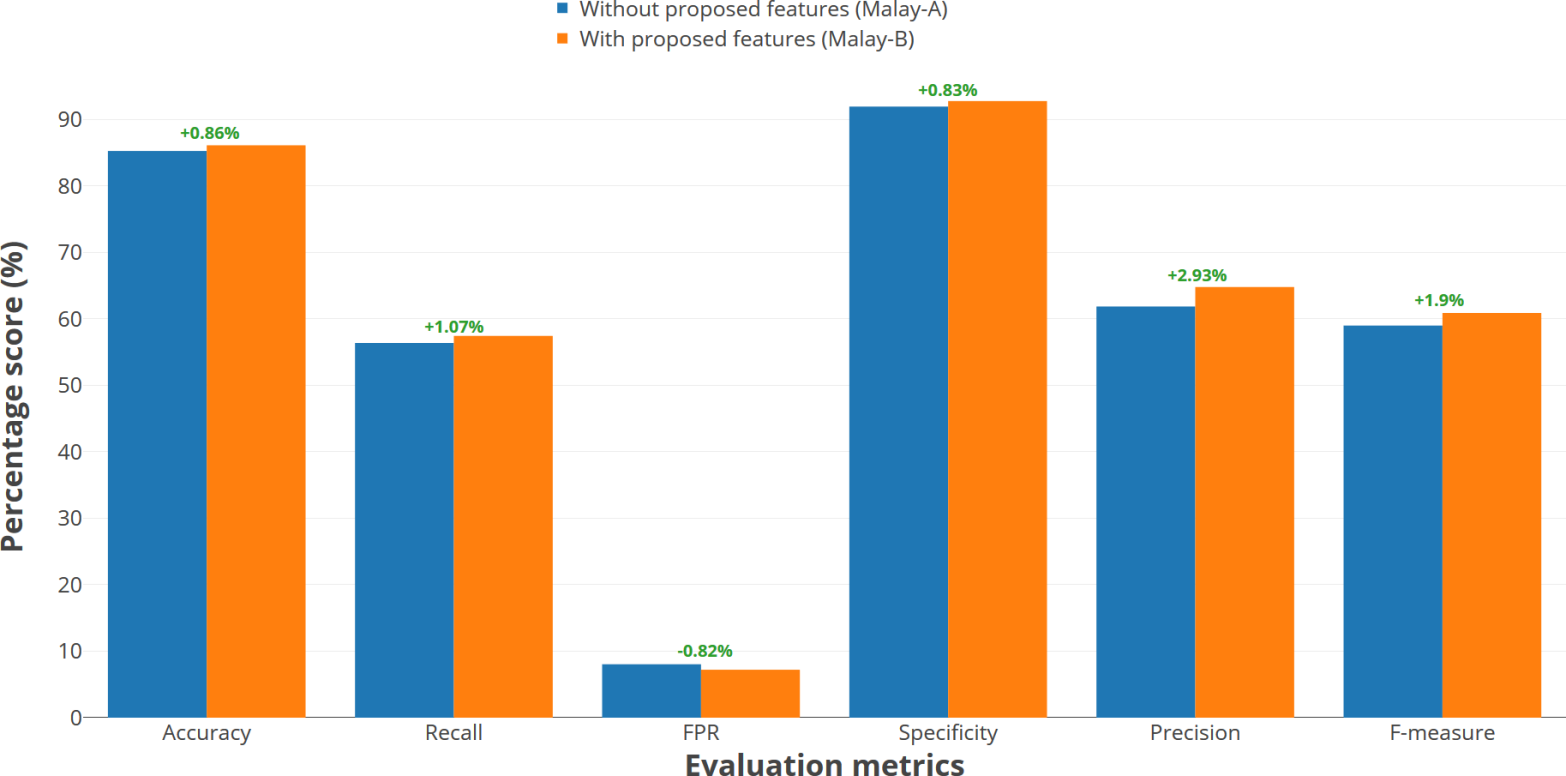
Percentage of score difference between Malay-A and Malay-B models.

The evaluation of the Malay dataset through the Malay-A and Malay-B models showed that the proposed features had also increased the accuracy rate of the detection by 0.86 per cent. The Malay-B model achieved 86.13 per cent detection accuracy with 57.45 per cent recall percentage. This implies that it is better and more precise in detecting opinion spams. The Malay-B had actually increased in percentage in terms of specificity, precision and f-measure—by 0.83 per cent, 2.93 per cent and 1.9 per cent respectively. The false positive rate also reduced in the Malay-B model due to the increment of the recall. The f-measure percentage showed that the model was more balanced in detecting the opinion spams and the normal opinions including the false positive and false negative opinions. This implies that the Malay-B model had increased in performance as a result of the implementation of the proposed features. Fig 10 shows the percentage of score difference between both Malay predictive models.

This study had aimed to detect opinion spams by using supervised boosting approaches on multilingual datasets. The analysis showed that the XGBoost is suitable as a boosting approach for detecting opinion spams in the English language dataset while the GBM Gaussian is suitable for detecting the opinion spam in the Malay language dataset. In looking at the multilingual models, it is deduced that the GBM Gaussian is suitable for detecting both the English and Malay opinion spams. Results had also highlighted that the proposed features used in the model of this study had increased the performance of the model in detecting opinion spams in multilingual datasets. Thus, the aim of this study is fulfilled.

## Conclusion

In this study, we proposed using supervised boosting approached that use statistical based features to detect opinion spams in multilingual datasets. In the comparative evaluation of the existing features using different boosting approaches, it was noted that the XGBoost performed best in detecting opinion spams in the English dataset due to its higher recall percentage. In terms of the Malay dataset, it was found that the GBM Gaussian outperforms other classifiers as it was able to balance the detection of normal and spam opinions, with a higher F-measure percentage. This study also performed a comparative evaluation of new statistical based features using supervised boosting approach on multilingual datasets. The new statistical based features along with the XGBoost achieved a detection accuracy rate of 87.43 per cent on English. Meanwhile, the detection accuracy achieved on the Malay dataset had increased to 86.13 per cent. This study shows that the proposed features had increased the effectiveness of the model in detecting opinion spams on multilingual datasets. This study also encourages more studies to use the boosting approaches to solve opinion spam detection problems.

### Limitations and future works

In the earlier part of this paper, it was noted that there is a lack of public dataset to be used for opinion spam detection of Malay language reviews. It is very important to have a gold standard dataset so that they are accessible and can be used publicly by other researchers. Besides that, there are few known works about opinion spam detection that specifically focussed on Malay language reviews and websites. Consequently, it restrains the conduct of any other research due to the lack of resources. Most studies have been focussing on analysing the works of opinions written in the English language thus it is high time that studies address this scarcity. Another recommendation is to expand on the works of opinion spam detection in the Malay language by using another approach for example, by incorporating Natural Language Processing into the model used. With this, other linguistic-based features can be generated and used to enhance the performance of the detection model. As a matter of reality, the Malay language is not a consistent language to be examined either because it consists of many types of accents derived from the various dialects of speakers or that the Malay used in reviews may also contain short forms and distinguished word structures. Based on this, it may be a good idea to consider the necessity of knowing the language and the meanings of the use of words and sentences by speakers so as to be able to understand the reviews written in Malay more precisely. In addition to that, the use of temporal and spatial based features may also need to be considered since it was found to be very reliable in detecting the English opinion spams as evidenced by Li, Chen [56]. Finally, it is hoped that collaboration between companies and organisations can be prolonged so as to provide mobile users and application users with a livelier and filtered set of data for future experiments.
